# Novel SMD Component and Module Interconnection and Encapsulation Technique for Textile Substrates Using 3D Printed Polymer Materials

**DOI:** 10.3390/polym15112526

**Published:** 2023-05-30

**Authors:** David Kalaš, Radek Soukup, Jan Řeboun, Michaela Radouchová, Pavel Rous, Aleš Hamáček

**Affiliations:** Faculty of Electrical Engineering, University of West Bohemia, Univerzitní 8, 301 00 Pilsen, Czech Republic; rsoukup@fel.zcu.cz (R.S.); jreboun@fel.zcu.cz (J.Ř.); mradouch@fel.zcu.cz (M.R.); rousp@fel.zcu.cz (P.R.); hamacek@fel.zcu.cz (A.H.)

**Keywords:** interconnection technique, encapsulation, e-textile, 3D printing

## Abstract

Nowadays, a range of sensors and actuators can be realized directly in the structure of textile substrates using metal-plated yarns, metal-filament yarns, or functionalized yarns with nanomaterials, such as nanowires, nanoparticles, or carbon materials. However, the evaluation or control circuits still depend upon the use of semiconductor components or integrated circuits, which cannot be currently implemented directly into the textiles or substituted by functionalized yarns. This study is focused on a novel thermo-compression interconnection technique intended for the realization of the electrical interconnection of SMD components or modules with textile substrates and their encapsulation in one single production step using commonly widespread cost-effective devices, such as 3D printers and heat-press machines, intended for textile applications. The realized specimens are characterized by low resistance (median 21 mΩ), linear voltage–current characteristics, and fluid-resistant encapsulation. The contact area is comprehensively analyzed and compared with the theoretical Holm’s model.

## 1. Introduction

Nowadays, there is a growing demand for the real-time monitoring of sports activities (heath rate, lactate, volume of oxygen VO_2_, acceleration, speed, cadence, and overall training load) [1] and the body’s vital [2] or environmental parameters (temperature, humidity) [3] with the aim of increasing safety, health protection, or comfort. E-Textiles provide the advantages of flexibility, light weight, and soft electronic function block integration directly into the textile, removing cables, bulky interconnections, and mechanical interfaces. Hence, the integration of electronics into everyday products, such as textiles, is becoming more common. The area of e-textile applications is continuing to expand, incorporating new materials, the integration of new technologies, and the optimization of production processes. E-textiles enable a wide range of applications with respect to health monitoring (e.g., edema [4] or apnea [5] monitoring, hydration [6], and temperature measurement [7]), maintaining a comfortable temperature [8,9,10], measuring mechanical strain [11,12] integrating safety LED lighting [13], and facilitating wireless communication by means of textile antennas [14]. Conductive patterns and sensors on textile substrates can be created by typical textile fabrication technologies, such as weaving [15,16], knitting [17,18], or embroidering (Table 1) using microwires [19,20] or electrically conductive polymer yarns [21,22,23,24], by the deposition of conductive polymer pastes [25], or by a combination of the selective patterning of Ag NPs and electroless Cu-plating [26].

However, conventional semiconductor components or evaluation circuits either in SMD (surface-mounted device) packages or mounted on printed circuit boards (PCB) cannot easily be substituted by conductive or semiconductive yarns and have to be placed and electrically contacted on the textile substrates. The majority of electrical interconnection techniques used in standard PCB production and the semiconductor industry are not suitable for textile substrates due to the high temperature of the production process and certain disadvantageous properties of the electrical materials, such as their insufficient flexibility, unsuitable dimensions, lack of wash resistance, and low degree of expansion compared to textiles (which can cause cracks during temperature shock changes), etc. The most common technology for assembly components in standard PCB production is nowadays fully automated reflow soldering. An effort has been made to transfer this technology to e-textile; however, due to textile substrate low-temperature resistance, dimension instability, large areas of character, and poor wettability, textile-based conductors create difficulties that limit the soldering in e-textile applications. There are other interconnection techniques that are more compatible with textiles (Table 2), such as sewing of electronic modules on flexible polyamide foil by conductive yarns [27,28], manual low-temperature soldering by bismuth solder [29,30], ultrasonic welding [31,32,33], resistance welding [34], and joining by conductive or nonconductive UV-cured and melt adhesives [35,36,37] conductive silver-plated hook and loop strips [25,36,38], snap fasteners [39,40,41], the 3D-printed press-fit socket using filaments filled by carbon [42], etc. However, all these techniques also have certain weaknesses, such as the instability of electrical contact resistance based on the gradual loosening of fibers of yarns of sewed contacts, increasing electrical resistance due to the silver tendency to peel off quickly out of hook and loop strips, the high electrical contact resistance of carbon-filled filaments for 3D printing, cost of adhesives, or the risk of some electrical components becoming damaged during ultrasonic welding interconnection process.

In view of this fact, there is a demand for a unique, versatile, low-cost interconnection technology to integrate both SMD components and PCBs, which provides contact resistance below 50 mΩ negligible to the electrical resistance of conductive yarns with an average resistance of 5 Ω/m, water-resistance, removing high-cost input materials and production devices with emphasis on the use of conventional textile production equipment. The interconnection of the electronic component to the substrate is not usually the final production step leading to reliable and stable electrical interconnection. E-textiles with high reliability, exhibiting electrical interconnections with good mechanical, chemical, and wash resistance, require further production process steps, such as additional encapsulation. Due to the fact that e-textile uses rigid SMD components or modules placed on the flexible or even stretchable textile substrate, all the mechanical stresses are concentrated in the contact area. The additional encapsulation materials move the mechanical stresses mainly to the textile substrate and also can provide strain relief features with a gradient toughness for the smooth changeover from the flexible textile substrate to the rigid contacted component materials. Besides the increase in mechanical resistance, encapsulation also provides resistance during the usage and maintenance to all environmental aspects, such as chemicals, water, and humidity. Nowadays, such techniques as glob top, dam-and-fill, gravity casting, or injection molding are used in commercial PCB manufacturing [45] and have also been experimentally tested in the area of e-textiles [27]. Nevertheless, such techniques require more production equipment and costs. For the reasons mentioned above, this study presents the comprehensive description and characterization of a novel thermo-compression interconnection technique, where the SMD components or PCB modules are assembled into the cavity of a 3D printed thermoplastic housing manufactured by fused filament fabrication (FFF); this housing is then placed on the textile substrate with the integrated conductive pattern and melted and cooled under continuous pressure by a heat-press machine. As a result, the electrical contact was created together with the encapsulation of the component, which fulfilled the above-mentioned criteria of low-resistance electrical contact, water resistance encapsulation, compatibility with standard manufacturing textile processes, and was commonly used and widespread production equipment.

## 2. Materials and Methods

### 2.1. Thermo-Compression Interconnection Technique

**Figure 1:**
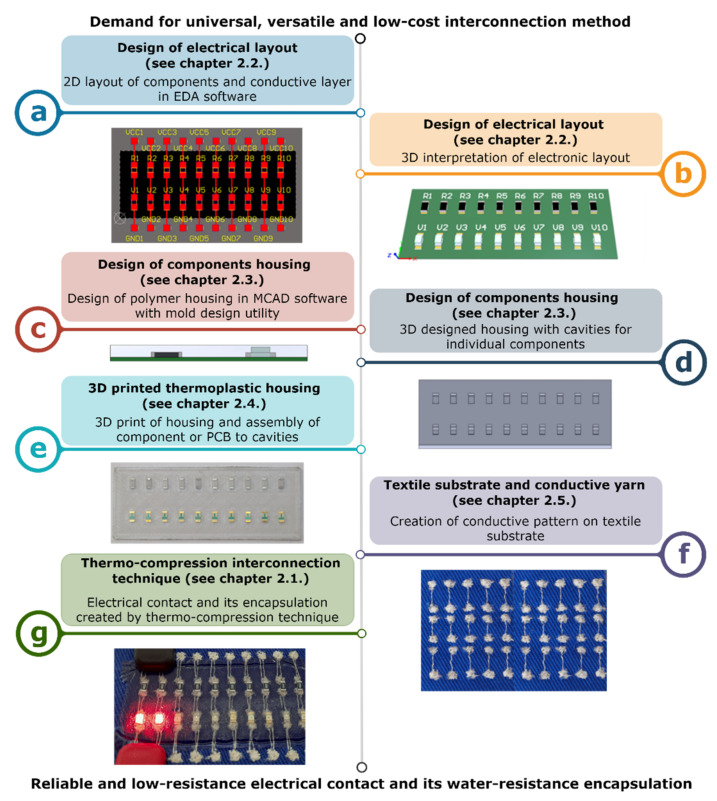
Process of interconnection by the thermo-compression technique. (**a**) the 2D layout of component and conductive layer in EDA software. (**b**) the 3D interpretation of electronic layout. (**c**) design of housing in MCAD software with mold design utility. (**d**) the 3D-designed housing with cavities for individual components. (**e**) the 3D print of housing and assembly of components of PCB to cavities. (**f**) creation of conductive pattern on textile substrate. (**g**) electrical contact and its encapsulation created by the thermos-compression technique.

The thermo-compression interconnection process (Figure 1) consists of several comprehensive design and production steps divided into two categories, which are described in detail in the next chapters: (i) electronic production (electrical layout design using EDA software, the design, and creation of the polymer housing); and (ii) e-textile production (preparation of the textile substrate, creation of the conductive pattern, and final interconnection). The electronic components are assembled directly into the polymer housing created, e.g., by the 3D printer or injection molding, so the process of electronic and e-textile production can be divided between appropriate manufacturers, thus making the production process more efficient.

The novelty of the presented technique lies in its combination of increased temperature and pressure to create electrical contact between an electronic component or PCB and a textile substrate and also in its encapsulation of the contact and components in a single production step (Figure 1g). A standard heat-press machine intended for the thermo-transfer of decorative pictures or patterns on clothes, for example, can be used. This type of heat-press machine is widely available and commonly used in textile manufacturing, which significantly decreases the costs of implementing thermo-compression interconnection technology in the production process. Heat-press machines for textile applications usually consist of a heated top plate and a bottom non-heated plate, which can be optionally equipped with an air membrane for component height compensation and pressure adjustment.

In this study, a Secabo TC5 Smart heat-press machine (Secabo, Wolnzach, Germany) was used for specimen preparation. A heat-press machine consists of a top planar rigid heated plate (dimensions of 38 cm × 38 cm and maximum temperature 225 °C) and a bottom non-heated air membrane. The air membrane enables differences in electronic component and textile heights to be balanced and the pressure of the thermo-compression process to be adjusted up to 600 g/cm^2^. The low-end heat-press machines are not equipped with an air membrane, and the pressure is adjustable only by a screw mechanism without pressure measurement. However, the dimensions of the common heat-press machines are ordinarily similar and adapted to the dimensions of t-shirts intended for the thermo-transfer of images. The thermo-compression profile consists of three parts: (i) heating to peak temperature; (ii) dwell time at peak temperature; and (iii) cooling below 50 °C. The cooling time depends on the dimensions, the level of thermal insulation, and the availability of active cooling by the heat-press machine. For the Secabo TC5 machine, the total process time for such textile applications is approximately 15 min and is not conditional on the number of realized electrical contacts. The total process time can be decreased using a custom heat-press machine with suitable dimensions and active cooling. In our study, the pressure during the whole process was adjusted to 600 g/cm^2^ (58.84 kPa), and a peak temperature of 175 °C was maintained for 90 s. These parameters are optimized for used thermoplastic materials and SMD components. The temperature varied from 165 °C to 195 °C. The final process temperature was chosen based on the melting level and the adhesion of the thermoplastic housing material to the textile.

**Figure 2:**
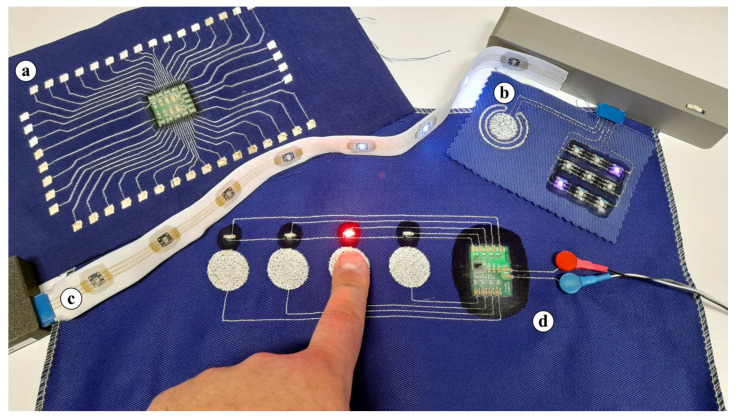
Specimens of e-textiles with integrated components by the novel thermo-compression interconnection technique. (**a**,**b**) show interconnections of PCB module on a cotton textile substrate. (**c**) shows PCB module on a stretchable textile ribbon. (**d**) SMD component and PCB module on a cotton textile substrate.

**Figure 3:**
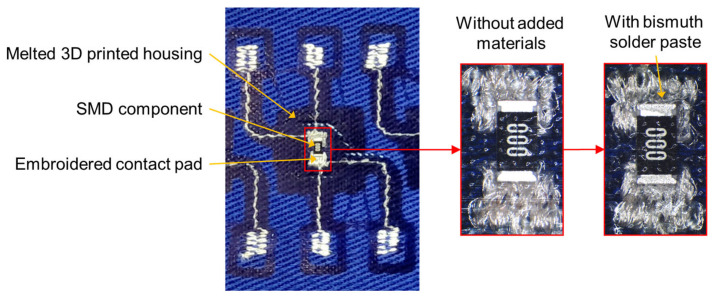
Detail of contacted resistors in a variant without added materials and with added solder paste.

The electrical contact is realized by two mechanisms, (i) the SMD component or the electronic module on PCB is pressed onto conductive pads located on the textile substrate (Figure 2), resulting in a mechanical contact without the creation of a metallurgical joint (Figure 3), but with the optional addition of a conductive filament or paste with metal particles to increase reliability and decrease electrical resistance, or (ii) a Bismuth-based low-temperature solder paste is applied on the pads and melted under the same conditions that are used for the thermo-compression of the housing material at a temperature above 160 °C (Figure 3). In either case, the component housing is melted and pressed onto the textile substrate, thus also encapsulating the component. The reliability of electrical contacts and their encapsulation can also be influenced by the orientation of the component in the heat-press machine. The orientation of the components facing (i) the heated top plate or (ii) the bottom non-heated plate with the air membrane of the heat-press machine was investigated. The reliability of created specimens was evaluated based on electrical resistance value and trend (the criteria for high-quality contact was 50 mΩ) during the exposition to maintenance in a washing and drying machine in accordance with ISO 6330:2012 “Home Washing and Drying Procedures for Textile Testing”. Specimens were washed at a 40 °C temperature, with a spin speed of 400 rpm, using 20 g of standardized detergent ECE type 3 without phosphates and dried for 60 min at 60 °C.

### 2.2. Design of Electrical Layout

Both SMD components and PCB modules can be contacted by the thermo-compression interconnection technique. The electrical layout on the textile substrate is designed with two limitations, (i) the contact pads spacing of the SMD components and PCB modules are dependent on the manufacturing technology used to create the conductive patterns on the textile substrate and have to be determined before the electrical layout (in the case of embroidered patterns, the spacing has to be larger than 1 mm), and (ii) a one-sided electrical design with a planar contacting surface must be used. The component spacing is limited by the housing dimensions after the thermo-compression process. More thermoplastic material in the area of the component requires more component spacing to preserve the flexibility of the textile. The two-part housing for components with a gradient toughness interface was designed in this study, so the minimum component spacing was 10 mm. The designed conductive trace width depends on the technology of conductive pattern creation. The trace width of the textile-based conductive patterns made by knitting, weaving, or embroidering depends on the used conductive yarns, so the trace width in the electrical layout is unimportant. In addition, the pattern realized by weaving and knitting is limited by the chosen textile weave because these technologies do not enable the creation of arbitrary patterns. Other design rules are the same as for the standard printed circuit boards. Two output files from Electronic Design Automation (EDA) software, such as Altium Designer, KiCad, OrCad PCB designer, etc., for the subsequent design and production processes, are required: (i) a DXF or PDF export of the conductive copper layer for the design of the conductive pattern on the textile substrate (Figure 1a); and (ii) a 3D interpretation of components and PCB in STEP or Parasolid format (Figure 1b).

### 2.3. Design of Component Housing

**Figure 4:**
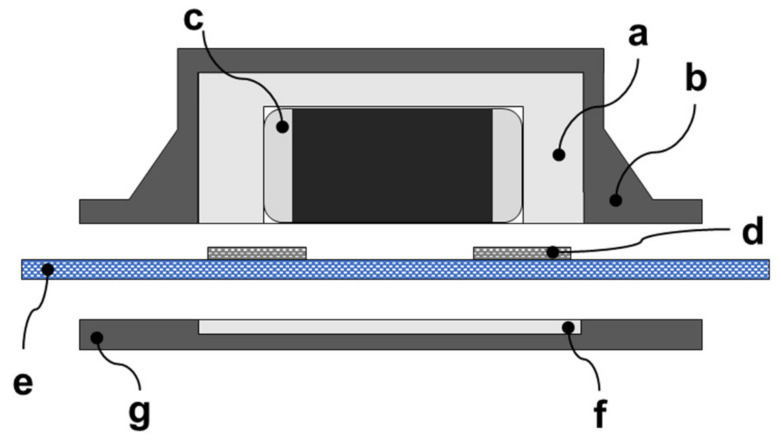
Topology and composition of the polymer housing with the SMD component for the thermo-compression interconnection technique. (**a**) the rigid part of the housing with the cavity for the component. (**b**) flexible flange. (**c**) SMD component. (**d**) contact pad embroidered with hybrid conductive yarn. (**e**) textile substrate. (**f**,**g**) planar layer of rigid and flex material placed on the reverse side of the textile substrate.

The component housing is designed using mechanical computer-aided design (MCAD) software, such as SolidWorks, AutoCAD, PTCCreo Parametric, etc., on the basis of a 3D interpretation of components and PCBs by the utility for mold design (Figure 1c). This utility is a usual part of MCAD software and enables both easy imprint creation (Figure 1d) and easy adjustment of the cavity scale for the assembly of components or PCBs in only a few steps. The dimensions of cavities depend on the tolerance of component production, the production techniques, and the complexity of the integrated electronics. Out of consideration for textile substrate flexibility, the reliability of the e-textile, and the stability of the electrical contact resistance, the topology and material composition of the housing have to be more complex. The main housing part for encapsulating the component (Figure 4c) is made of rigid material (Figure 4a), which provides mechanical and chemical resistance and the non-flexible fixation of the component in the area of the textile substrate’s contact pads (Figure 4d). The other part of the housing is made of flexible material (Figure 4b) and creates a flange with a gradient toughness for the smooth changeover from the textile substrate (Figure 4e) to the contacted component. A flexible material can optionally be applied as a thin selective encapsulation layer for a conductive pattern in order to increase wear-out resistance. Both materials—rigid and flexible—are also designed in the thin planar layer intended for placement on the reverse side of the textile substrate (Figure 4f,g), thus resulting in a component that is completely encapsulated from both sides and, therefore, in an increase in mechanical and chemical resistance.

Both SMD components and PCB modules can be contacted by the thermo-compression interconnection technique; however, this study is focused on specimens with individual “zero-ohm” chip resistors in SMD (surface mount device), package type 1206, with dimensions 3.2 mm × 1.6 mm × 0.6 mm. The dimensions of the housing cavity were larger than the SMD resistor by 0.1 mm along all axes for effortless component assembly, and the external dimensions of the rigid housing part were 4.3 mm × 2.7 mm × 1.2 mm. The external dimensions are designed according to a minimum extrusion width of 0.45 mm and 3D printed material consumption because the flexibility of the textile decreases with increasing thermoplastic material in the area of components. The quality of electrical contacts was evaluated mainly on the basis of the measurement of electrical resistance. The typical electrical resistance of electrical contacts created by the thermo-compression technique is in tens of mΩ. Thus, an electrical component with a resistive character, whose tolerance is comparable with electrical contact resistance, cannot be used for precise characterization. “Zero-ohm” resistors with a median electrical resistance of 8 mΩ and negligible resistance tolerance were used in this study. The absolute value of “zero-ohm” resistors also does not affect the measurement range and, therefore, the measurement current applied to specimens by the 4-wire resistance measurement technique.

### 2.4. 3D Printed Thermoplastic Housing

The additive FFF 3D printing of polymer materials is a widely-used technique, especially in rapid prototyping. 3D printing is naturally becoming increasingly common in high-mix, low-volume (HMLV) commercial production because of its low price and the high availability of 3D printers and polymer filaments, the possibility of multicolor printing, and the availability of biodegradable, chemically-resistant, and mechanically resistant 3D printing materials. On the other hand, low-temperature resistance, the necessity of final treatment to achieve a smooth surface without visible 3D-printing layers, and the time-consuming process of 3D printing extensive series still limit FFF technology. Nevertheless, the 3D printing of polymer housings for components or electronics modules on PCB is independent of the type and manufacturer of the 3D printer. In view of the fact that the 3D-printed housing with the assembled components (Figure 1e) is melted and cooled under continuous pressure by a heat-press machine, any slight defects of such a 3D-printed object caused by the usage of a low-cost 3D printer, the imperfect storage of 3D printing materials, or inappropriately set 3D printing parameters will be negligible. The housings can be printed in a wide range of colors and shapes designed to make component and electronic module measurements.

Materials for the creation of polymer housings were selected on the basis of a previous study [46] focused on the thermal and dielectric properties of 3D-printed polymers and the material properties described by the manufacturers (e.g., UV stability, chemical resistance, bio-compatibility, and environment-friendliness) [47,48,49,50]. A rigid material based on acrylonitrile styrene acrylate (ASA) and flexible elastomers based on thermoplastic polyurethane (TPU) were selected for further analysis. The filaments were extruded by a Prusa MK3s 3D printer (Prusa Research, Prague, Czech Republic) through a nozzle with a diameter of 0.4 m. A flat polyetherimide (PEI) surface was used on the heat bed. When the filament adhered insufficiently to the PEI surface, the special adhesive Magigoo (Thought3D Ltd., Paola, Malta) was used. The thickness of one printed layer was set to 0.2 mm. All samples were printed with 100% infill.

### 2.5. Textile Substrate and Conductive Yarn

**Figure 5:**
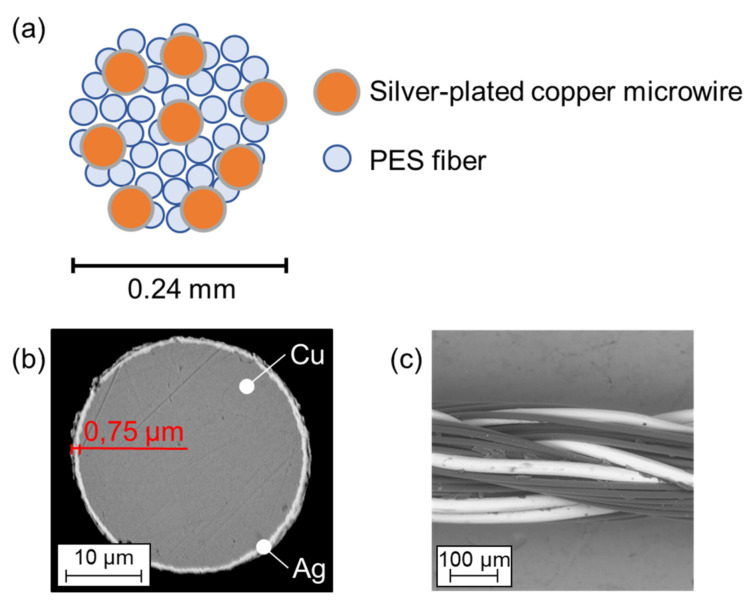
(**a**) Cross-sectional model of hybrid conductive yarn. (**b**) Analysis of silver-plated copper microwire by SEM. (**c**) longitudinal view of hybrid yarn HI-COND art. 74 (8× Cu/Ag microwire + polyester yarns).

The selection of textile substrates used for thermo-compression depends on their temperature resistance. Substrates composed, e.g., of natural silk, synthetic polyamide, or polypropylene fibers, are not suitable for high-temperature exposition (above 150 °C). Hence, textile specimens were prepared from 100% cotton (specific weight 243 g/m^2^) with twill fabric weaves. The used cotton twill weave is dimensionally stable and suitable for embroidered patterns with sufficient design repeatability. The conductive pattern was prepared by a Bernina QE750 conventional embroidering machine using a low-resistance hybrid conductive yarn (Figure 1f) consisting of standard polyester (PES) fibers with a diameter of 14 µm and 8 conductive silver-plated copper microwires (Figure 5a,c) with a diameter of 30 µm manufactured by Clevertex (Table 3) [51]. The microwire’s silver layer is created by electrochemical deposition on 8 mm-wide copper wires and subsequently manufactured by drawing to the required diameter; the final silver layer is 0.75 µm thick (Figure 5b). In this study, the hybrid yarns manufactured by Clevertex were selected because of their low electrical resistance compared to metalized polymer yarns and polymer conductive yarns and the limited flexibility of patterns created by the deposition of conductive pastes. However, the selection of conductive yarns is limited by their temperature resistance.

### 2.6. Electrical Characterization of Contact Resistance

**Figure 6:**
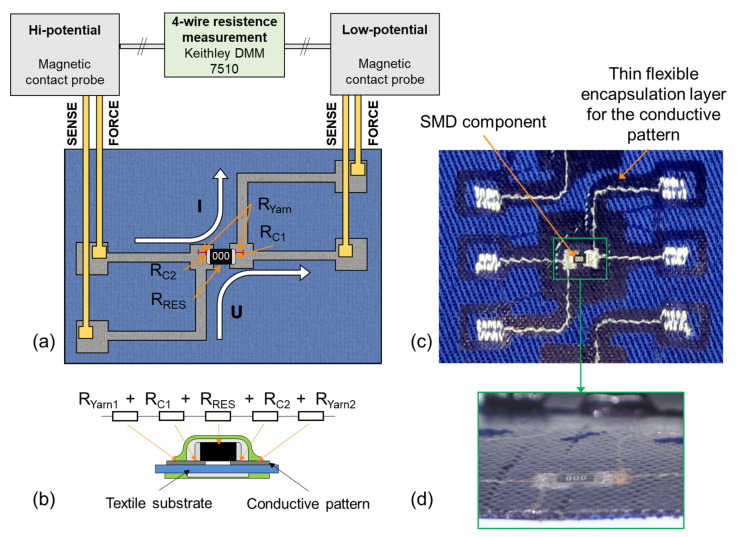
(**a**) pattern design and the principle of 4-wire resistance measurement. (**b**) sum of measured resistances. (**c**) specimen fabricated by the thermo-compression technique. (**d**) side view of encapsulated SMD component.

Specimens of SMD resistors contacted on the textile substrate were characterized electrically by the 4-wire measurement of resistance and voltage–current characteristics. The conductive pattern was designed for 4-wire measurement by magnetic contact probes (Figure 6a,c).

The electrical resistance was measured by the 4-wire method using a Keithley 7510 multimeter (Tektronix Inc., Beaverton, OR, USA) in the 1 Ω and 10 Ω range with a constant test current of 10 mA. The precision of the multimeter was 0.1 μΩ. As depicted in Figure 6a, the test current I is applied from the “Force” terminal to the contacted resistor, and the “Sense” probes detect the voltage drop. Unlike the conventional 2-wire method, the test current does not flow through the same terminal where the voltage drop is detected; hence, this method eliminates the resistance of measuring probes and cables and the resistance of conductive traces on the textile as well. Unique contact fixtures with spring probes and magnetic fixation were developed for the purpose of contacting the test objects. Because the SMD components are fully encapsulated, the resistance measurement of individual contacts is not possible (Figure 6b). Hence, the value of the measured resistance consists of five constituents, the contact resistances R_C1_ and R_C2_, 2 × R_Yarn_, and the resistance of the “zero-Ω” resistor R_RES_ with a real resistance of approximately 8 mΩ (Equation (1)). The 4-wire terminals were not connected to the specimens directly in the area of the electrical contact between the embroidered contact pad and the SMD resistor but ended at the edge of the embroidered contact pad, so the measured resistance also consisted of the yarn resistance R_Yarn_ between the electrical contact and conjunction of the 4-wire method. The dimensions of the embroidered contact pads were 2 mm by 2 mm with 1.2 mm spacing. The length of the SMD resistor was 3.2 mm, so approximately 0.7 mm per each side of the hybrid conductive yarn was included in the measured resistance equal to 3.99 mΩ, because the electrical resistance of hybrid conductive yarn with eight copper silver-plated microwires is 2.85 Ω/m.

R_C4w_ = R_Yarn1_ + R_C1_ + R_RES_ + R_C2_ + R_Yarn2_ (Ω) (1)

In the ideal case, any electrical contact should be characterized by a linear voltage–current dependence. Nevertheless, the realization and use of electrical contact in real conditions can negatively influence this linearity by, for example, (i) the presence of oxide films or impurities, (ii) a contact potential at the contact metals’ interface, or (iii) a temperature-dependence caused by the current flow. Measurement was performed by a Keithley SMU 2612B two-channel source meter (Tektronix Inc., Beaverton, OR, USA) using DC sweep voltage analysis with a 1 mV voltage increment up to the device current limit of 1.5 A. The test pattern and contact probes were the same as for the 4-wire resistance measurement, so the difference was only in the measurement procedure. The voltage source is connected from the “Force” terminal to the contacted resistor, and the “Sense” probe detects the development of the voltage drop and gives feedback to the source regulator. The current is measured in the “Force” terminal’s circuit.

### 2.7. Theoretical Model of Contact Resistance

The electrical contacts are, in many cases, created by the constriction of two solid objects which can conduct the electrical current. The contact area is not formed by one compact area, as the electrons flow through so-called α-spots, whose sum of individual spot areas is in many cases distinctively smaller than the theoretical contact area, a situation, which results from, for example, the presence of oxide films or impurities and the hardness of solid contact materials, or more precisely, their ability to resist deformation caused by the constrictive force. The electrical contact phenomena and the calculation of resistance are described in several theories derived by, e.g., Sharvin [52], Greenwood [53], and Holm [54,55,56].

**Figure 7:**
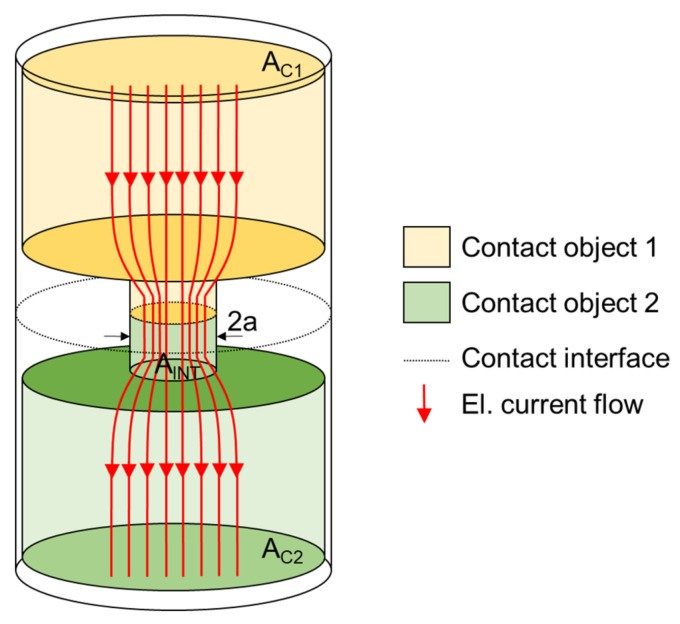
A theoretical model of electrical contact.

The calculation of electrical DC contact resistance in Holm’s theory can be simplified by employing one circular contact area corresponding with the area of α-spots (Figure 7). Two circular electrical contact materials with areas A_C1_ and A_C2_ and different electrical resistivities ρ_C1_ and ρ_C2_ are constricted together, creating an interface for electrical current flow with an area A_INT_ of radius a. The formula (Equation (2)) assumes (i) the presence of no oxide films or impurities, (ii) no deviation of current flow in the axial direction, and (iii) the solid contact objects having infinite dimensions to the current flow [55,56].

R_C_ = (ρ_C1_ + ρ_C2_)/4a (Ω) (2)

The electrical contact area is significantly affected by the contacting technique and can be in the range of tenths of µm^2^ for wire-bonded contacts up to areas of mm^2^ for soldered contacts. Wire-bonded contacts are commonly used in semiconductor components and represent the fact that even very small contact areas can be used for current flow and the high-volume production of electronics. Standardly-used wires have a diameter from 12.5 µm to 75 µm, and the contact area is in hundreds of µm^2^ [57,58,59]. Considering the 30 µm diameter of silver-plated copper microwires in the hybrid conductive yarn and surface impurities in the form of silicone or paraffin lubrication for easier yarn fabrication, similar contact areas for thermo-compressed and wire-bonded contacts are expected.

### 2.8. Quality of Encapsulation

**Figure 8:**
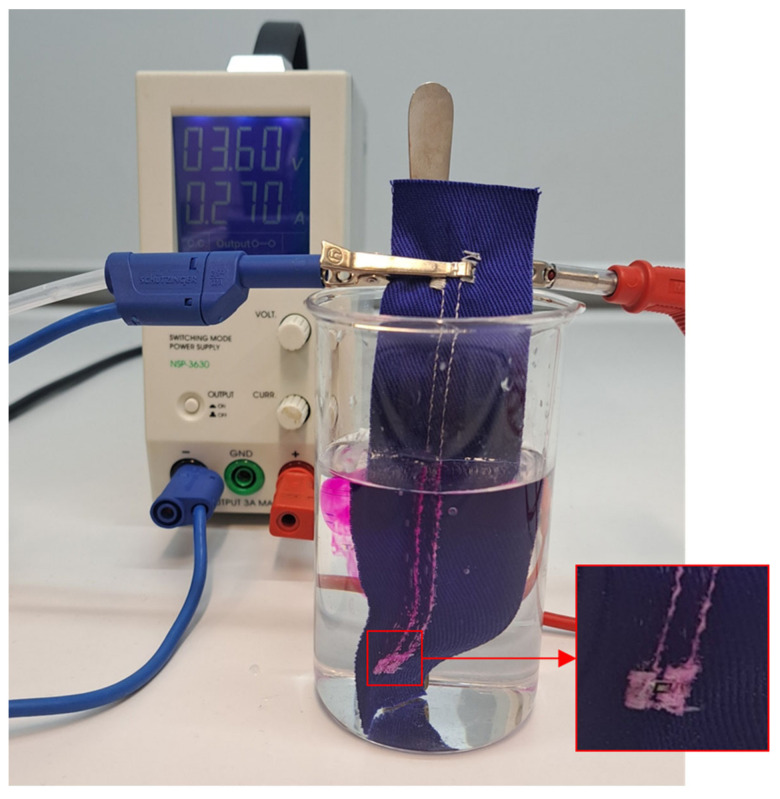
Specimen of a non-insulated soldered SMD component in a solution of sodium chloride and phenolphthalein.

The characteristics of electrical contacts are extremely important. However, the encapsulation of the contacted component is essential for the long-term reliability and stability of the e-textile product during its use when it is exposed to such influences as increased humidity, washing and drying cycles, sweat, cyclical mechanical bending, stretching, etc. The impermeability and compactness of the encapsulation were tested according to standard EN 60851-5, which focuses on the electrical testing of insulation continuity. Specimens were immersed in an electrolytic solution (1 L) of sodium chloride (2 g) and phenolphthalein (30 g) and connected to the cathode of the voltage source. Sodium hydroxide, which is highly alkaline, is produced by electrolysis on the cathode and, thus, increases the pH of the solution. The change in pH interacts with phenolphthalein and changes the color of the solution in the area of a defect. Simultaneously, gaseous hydrogen is released on the cathode and can be observed in the form of bubbles (Figure 8).

## 3. Results and Discussion

### 3.1. Component Orientation in the Heat-Press Machine

**Figure 9:**
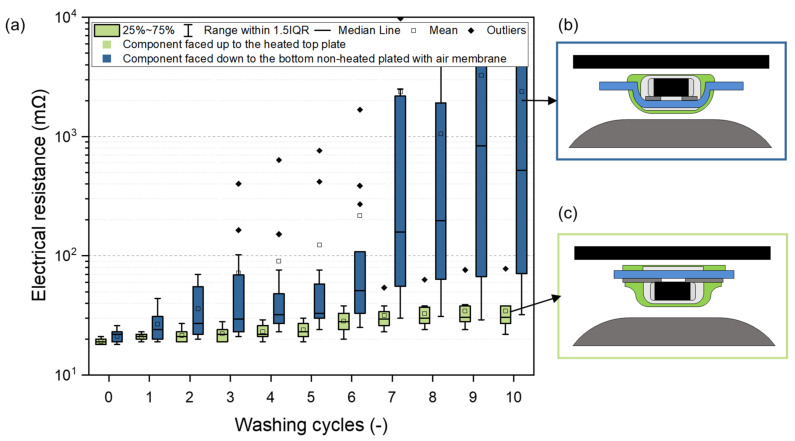
The influence of component orientation in the heat-press machine on (**a**) electrical resistance, (**b**,**c**) the profile of the e-textile and encapsulation in the area of the electronic component after the thermo-compression process.

Two types of orientation of the electronic component in the heat-press machine were tested, (i) facing the heated top plate (i.e., facing up) and (ii) facing the bottom non-heated plated with the air membrane (i.e., facing down). The specimens for this test were prepared without added materials because the orientation of the component has no influence on contacts comprising a metallurgical contact with the addition of solder alloy. Negligible differences in electrical resistance were observed after manufacturing. Nevertheless, the influence of orientation had a considerable impact on resistance stability during maintenance cycles (washing and drying). The textile substrates and yarns relax and change their dimensions during the thermal cycles. The tension of yarn exposed to increased temperature decreases after cooling to room temperature. This phenomenon was also observed in the study focused on sewing contacts [60]. As a result, a lower electrical resistance was measured when the contacted component was placed in the heat-press machine facing upward (Figure 9a), i.e., the textile substrate and planar layer were in direct contact with the bottom non-heated plate. Thus, in this case, the textile substrate is pressed against the top heated plate and fits closely to the polymer housing with the SMD component and also affects the electrical contact by natural textile tension. The textile substrate is fixed in the strained position after the thermo-compression process and solidification of the polymer housing. Simultaneously, the shape of the e-textile after the thermo-compression process with orientation facing up to the top heated plate decreases the component tear-off risk; based on the fact, the orientation of the component facing up to the heated plate increases the long-term reliability and stability of the e-textile made by the thermo-compression technique. The median resistance during the washing and drying test did not exceed 38 mΩ in this case. In contrast, the resistance of specimens facing the air membrane grew to almost 10 kΩ (Figure 9).

### 3.2. Specimens with Added Solder Materials for Electrical Conductivity Enhancement

**Figure 10:**
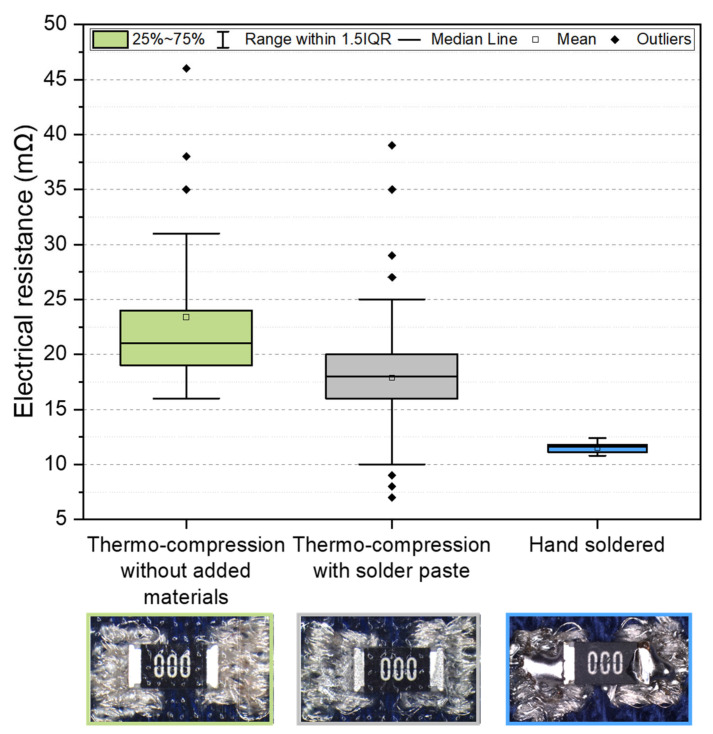
The electrical resistance of specimens prepared using different principles of electrical contact creation.

**Figure 11:**
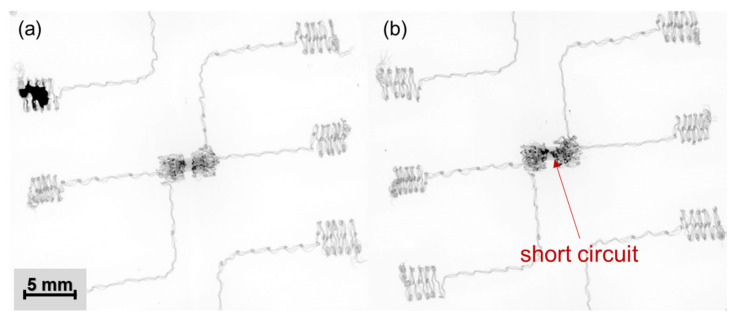
X-ray images of electrical contacts created by thermo-compression with (**a**) an appropriate and (**b**) an excessive amount of solder paste.

In total, 200 “face-up” specimens were prepared (50% without added materials and 50% with added solder paste). Figure 10 shows the sum of electrical resistance for electrical contacts and a zero-ohm resistor (8 mΩ). A higher electrical resistance with a median value of 21 mΩ was observed for specimens without added materials (Variant 1), in contrast to 18 mΩ for specimens with solder paste (Variant 2) reflowed during the thermo-compression process. The standard deviation was 8.63 mΩ for Variant 1 and 5.23 mΩ for Variant 2. The median resistance of reference specimens prepared by hand soldering was 11.6 mΩ with low variance (a standard deviation of 0.47 mΩ). However, using thermo-compressed electrical contacts with the addition of solder alloy entails some disadvantages: (i) the solder paste has to be deposited precisely because an excess can cause short circuits (Figure 11); (ii) only low-temperature bismuth-based solder paste can be used; (iii) flux residues are encapsulated altogether with the component; and (iv) poor wetting of the most textiles with solder.

### 3.3. Voltage–Current Characteristic

**Figure 12:**
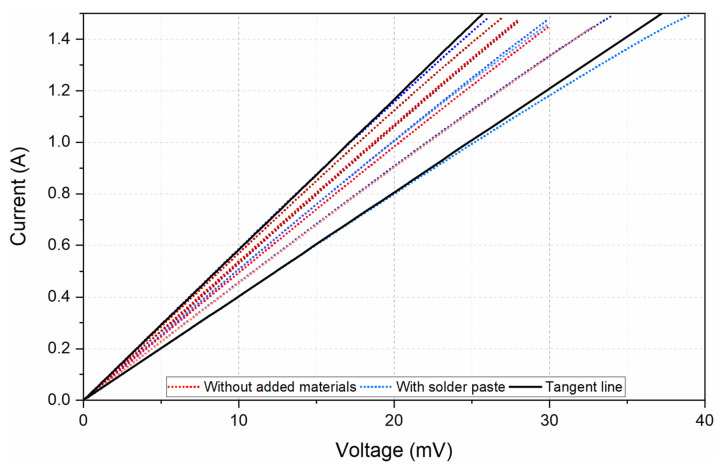
Voltage–current characteristics of electrical contacts prepared by the thermo-compression interconnection technology.

The median electrical resistance of contacted SMD zero-ohm resistors is 21 mΩ for contacts without added materials and 18 mΩ for contacts with added solder paste; hence, the specimens voltage drop was only in the range of 28 mV to 38 mV when the current limit 1.5 A was reached (Figure 12). The electrical resistance of hybrid conductive yarn with eight copper silver-plated microwires is 2.85 Ω/m; therefore, the power dissipation of the electrical contact created by the thermo-compression interconnection technique is insignificant in comparison with that for conductive yarn. An electrical current of 1.5 A represents a power dissipation of 641 mW per 10 cm of conductive yarn; thus, greater current flows and voltage drops with respect to electrical contacts are not expected for e-textile applications. At a voltage up to 40 mV, the electrical breakdown of oxide films and impurities was not observed; the voltage–current characteristic was linear for both mechanisms of electrical contact creation (metallurgical and pressed contact). However, the voltage–current characteristic exhibited a slight curve due to the temperature dependence of the electrical contact resistance [56]. In this case, the current density increases with current flow, and thus, the temperature of the electrical contact rises due to the generated Joule heat. Thus, higher electrical contact resistance implies higher power dissipation and a higher contact temperature. In view of this fact, contacts with a higher voltage drop and, therefore, greater power dissipation exhibit more curvature in their voltage–current characteristics (Figure 12).

### 3.4. Electrical Contact Area Analysis

**Figure 13:**
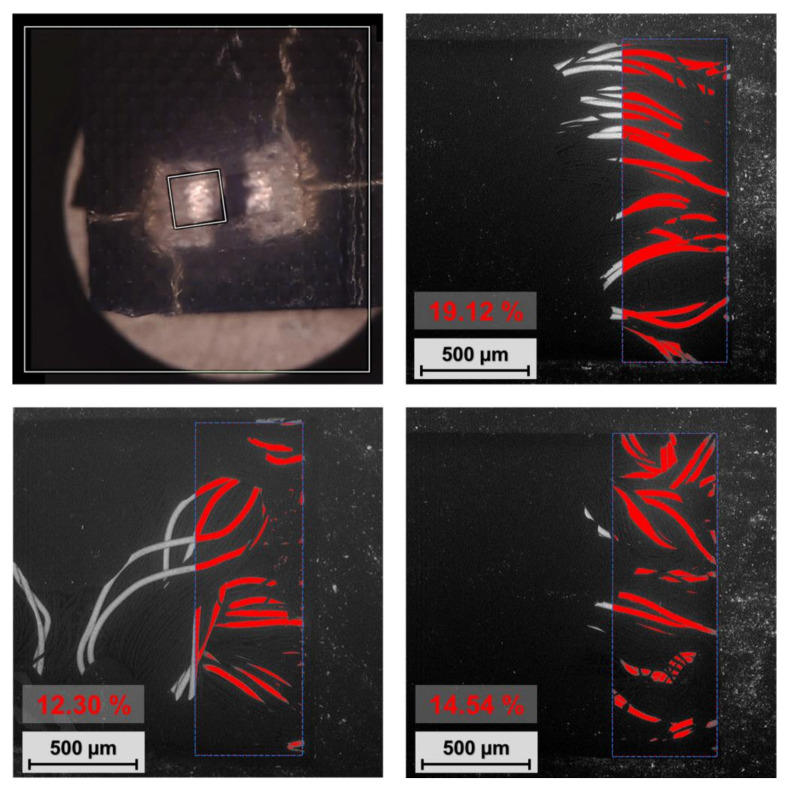
Analysis of the maximum theoretical contact area consisting of hybrid conductive yarn microwires.

The electrical contact area is an important factor with respect to optimizing embroidered conductive contact pad topology and thus increasing electrical contact stability and reliability, as well as defining the maximum current flow for admissible warming. The specimens for contact area analysis were prepared by the standard thermo-compression process and employed a 3D-printed housing topology without additional materials, such as solder paste. SMD components in the chip package type 1206 were integrated and subsequently unencapsulated and taken out after the manufacturing process; hence, the pressed embroidered contact pads were uncovered and subsequently analyzed by a Phenom PRO X Scanning Electron Microscope (Themo Fisher Scientific, Waltham, MA, USA). The dimensions of the component leads were 1.55 mm by 0.5 mm (0.775 mm^2^); this area is marked in Figure 13 by a blue dashed line. The maximum contact area formed by visible microwires of hybrid conductive yarn was determined by visual analysis and found to be in the range of 12.30% to 19.12%. On this basis, the theoretical (i) contact resistance (Equation (2)) (Table 4) and (ii) sum of measured resistance (Equation (3)) were calculated. Hence, the resistance measured by the four-wire method consists of five main parts R_Yarn1_, R_Yarn2_, R_C1_, R_C2_, and R_RES_, whose sum can be theoretically only 12.37 mΩ (Equation (3)).

R_C4w_ = R_Yarn1_ + R_C1_ + R_RES_ + R_C2_ + R_Yarn2_ = 1.99 + 0.189 + 8 + 0.189 + 1.99 = 12.37 (mΩ) (3)

**Figure 14:**
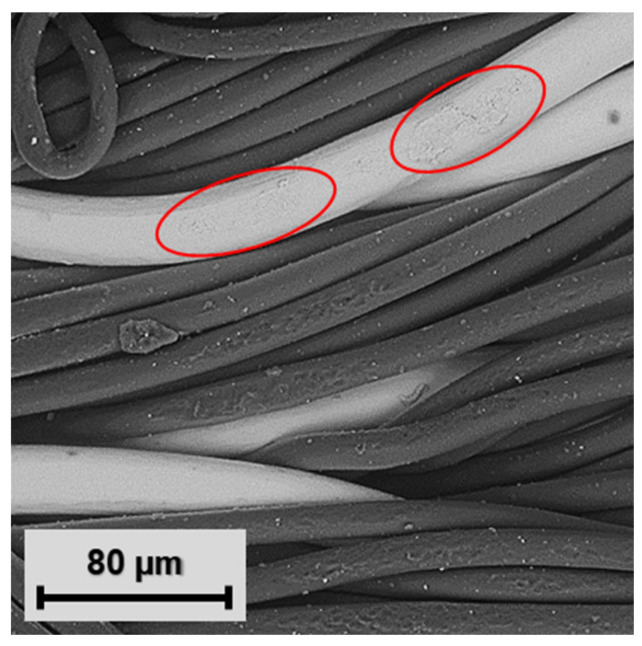
The contact areas of microwires.

However, the real contact area for current flow is influenced by (i) oxide films, (ii) surface impurities (microwires are lubricated by silicone or paraffin during the formation of yarn), (iii) the fact that the circular cross-section and material hardness of the microwire do not enable the creation of an α-spot across the whole diameter, but only in the tangential area, and (iv) the fact that not all visible microwires poke up from the embroidered pad and create the contact interface. In view of these points, the real measured electrical resistances of specimens are higher than theoretical values. The average electrical resistance of specimens after the manufacturing process was 21 mΩ. The real individual contact resistances R_C1_ and R_C2_ were 4.5 mΩ (Equation (3)), so according to Equation (4), if the contact materials are Ag for hybrid conductive yarn (0.016 × 10^−6^ µΩm) and Sn for leads of the SMD resistor (0.115 × 10^−6^ µΩm), the circle contact area radius is 7.27 µm, making a contact area of 166.4 µm^2^ (Table 5). The contact area in real electrical contacts is separated into clusters of small contact spots. These spots are unevenly distributed in the embroidered conductive pads and placed at the tangents of microwires, where slight deformation can be observed (Figure 14). However, the area of microwire deformation is not equal to the contact area for current flow with respect to surface impurities and oxide films; thus, evaluation of the real contact area given only by optical analysis is still quite complicated and imprecise.

R_C_ = (ρ_C1_ + ρ_C2_)/4a → a = (ρ_C1_ + ρ_C2_)/4 × R_C_ = (0.016 × 10^−6^ + 0.115 × 10^−6^)/(4 × 4.5 × 10^−3^) = 7.27 (μm) (4)

**Figure 15:**
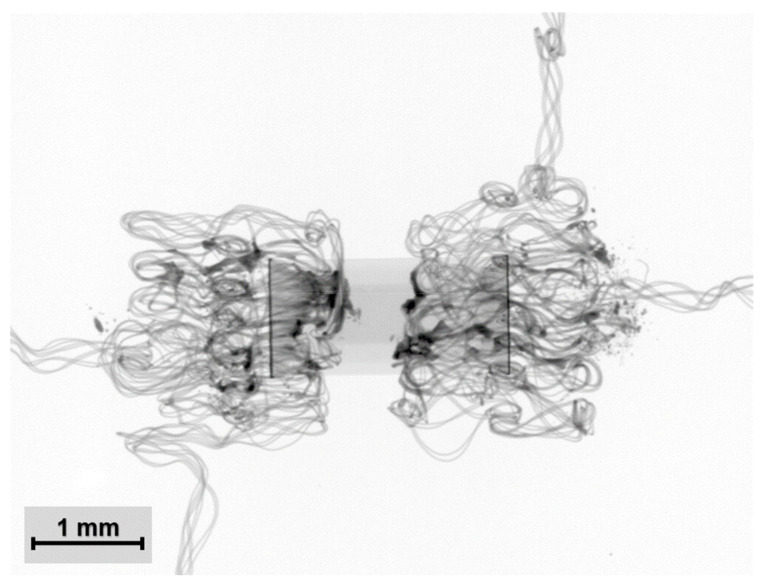
X-ray image of SMD resistor contacted by the thermo-compression technique with solder paste addition.

The contact area of specimens with added solder paste is also negatively affected by the oxide films and impurities because it should be much larger than in the case of specimens without added materials. The solder paste can pass into gaps in the embroidered pad, and the microwires, which do not poke up from the pad, can become wetted by the solder. Nevertheless, in our study, the median electrical resistance was 18 mΩ. This implies that the wetting of microwires is strongly affected by oxide films and impurities (Figure 15). Thus, surface treatment by plasma or pre-washing, as well as the addition of material with metal particles, could increase the number of contact spots and decrease the electrical resistance of hybrid conductive yarn. A similar effect could be achieved by changing the embroidered pad pattern and employing sparse sewing to increase its elasticity. Thus, the electronic component could be pushed into the pad, and conductive yarn microwires, which do not poke up from the pad, could be contacted.

### 3.5. Optical Inspection of Encapsulation

**Figure 16:**
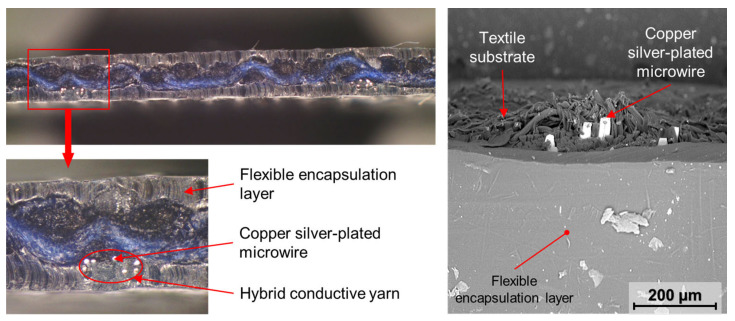
Optical inspection of textile specimen cross-section.

**Figure 17:**
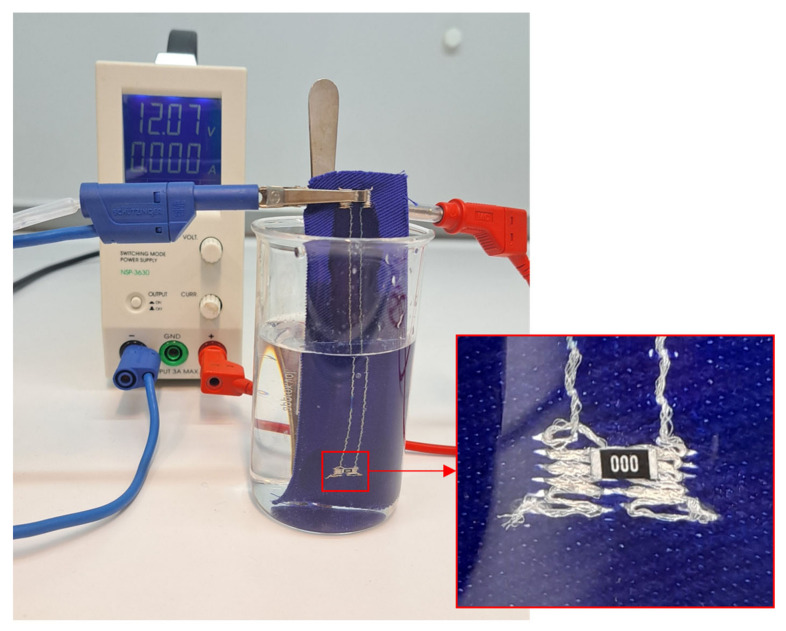
Test of insulation by immersion in a water solution of sodium chloride and phenolphthalein.

The water resistance of electronic component encapsulation influences the stability and reliability of specimens during exposure to, for example, increased humidity and maintenance in automatic washing and drying machines. The water that penetrates into encapsulation can make a micro-climate in the area of electrical contact, thus oxidizing metal materials in this area. Not only the electronic components themselves but also the conductive patterns specially prepared by embroidering techniques require additional encapsulation. The embroidered pattern protrudes above the textile substrate, so encapsulation increases scuff resistance. The results of the optical inspection of the encapsulated conductive pattern using a Phenom PRO X scanning electron microscope and an Olympus SZX10 stereomicroscope (Evident Corporation, Tokyo, Japan) are shown in Figure 16. The quality of insulation was verified by specimen immersion in a water (1 L) solution of sodium chloride (2 g) and phenolphthalein (30 g). No defect or micro-crack was detected (Figure 17).

## 4. Conclusions

A novel thermo-compression interconnection technique in which electrical contact is established together with its encapsulation in one production step was proposed, tested, and investigated. In contrast to currently-used interconnection techniques for e-textiles (ultrasonic and resistance welding, bonding by conductive or non-conductive adhesive, etc.), this novel thermo-compression technique (i) uses low-cost and multipurpose production devices, such as a 3D printer and a heat-press machine, which are commonly used in the textile industry, (ii) reduces the number of required production steps and thus costs, and (iii) is suitable for SMD components and also electronics modules on PCB. On the other hand, the use of conventional heat presses for textile applications increases the interconnection process time, although this time does not depend on the number of created contacts, which is only limited by the dimensions of the heat-press machine. The total process time can be significantly decreased by using a custom heat-press machine with active cooling. Hence, the thermo-compression interconnection technique is currently more suitable for high-mix, low-volume manufacturing, and small-to-medium-size enterprises (SMEs), which predominate in the E-textile area. In view of these facts, the developed interconnection technique could contribute to the spread of E-textiles among ordinary consumers.

The impact of the two possible orientations of components in the heat-press machine and the technique of electrical contact creation (without added materials and with a low-temperature solder paste applied on conductive pads) on electrical resistance was investigated. Specimens without added materials were strongly influenced by their orientation in the heat-press machine. The “face-up” orientation of the electronic component (i.e., facing the top heated plate) decreased the electrical resistance and increased stability under washing and drying cycles. Contacts with solder alloy were characterized by moderately lower electrical resistance (median R = 18 mΩ); however, the need for the precise deposition of the solder paste, the risk of short circuit creation, and the encapsulation of components along with the residues of flux are disadvantages. Despite the use of different mechanisms of electrical contact creation, the voltage–current characteristics of the metallurgical (with the addition of solder paste) and pressed (without added materials) contacts were linear up to 1.5 A. The electrical breakdown of oxide films and impurities was not discovered in this range.

The efficiency of the encapsulation of the electronic component and the conductive pattern was examined. The encapsulation of the embroidered conductive pattern can increase scuff resistance, emerging, for example, during cyclic mechanical stress in washing and drying machines. Water resistance was verified by the immersion of samples in a solution of sodium chloride and phenolphthalein while connected to a voltage source; no encapsulation defects or micro-cracks were detected.

In accordance with Holm’s theoretical model of electrical contact, the contact areas of specimens without added materials were examined and compared with theoretical values. Less than 20% of the component lead area was formed by conductive microwires (148,000 µm^2^). However, oxide films, impurities, hardness, and different height placements of microwires in the embroidered pad significantly decrease the electrical contact area. In fact, the area of electrical contact is in the hundreds of square micrometers (166.4 µm^2^), similar to that for wire-bonded contacts.

An increase in the number of electrical contact spots, and thus, a decrease in electrical resistance, could be achieved by surface treatment aimed at eliminating oxide films and impurities by adding material with metal particles, such as conductive filaments for 3D printing, which do not contain flux and do not require any special devices or production steps, and, where possible, by adjusting the percolation threshold and thus eliminating the possibility of short circuits.

## Figures and Tables

**Table 1:** Comparing standard textile technologies for creation of conductive patterns in e-textiles.

Technology	Advantages	Disadvantages
Weaving	- Flat profile - High density of patterns	- Determined patterns (plain, twill, and atlas weave) - Not suitable for rapid prototyping - Material demanding sampling
Knitting	- High flexibility and stretchability - Option of 3D structure creation	- Determined patterns (weft and wrap knits) - Low dimension stability
Embroidering	- Fully customizable pattern - Integration after substrate creation	- Pattern sticks out the substrate (lower-abrasion resistance)

**Table 2:** Comparing standard technologies for interconnection of SMDs or electronic modules on textile substrates.

Interconnection Technology	Advantages	Disadvantages
Soldering [29,30]	- Established and scalable process	- Poor wetting of most textiles with solder - High process temperatures can damage textiles - Contamination deterioration by flux residues
Adhesive bonding [37,43,44]	- Simultaneous realization of electrical and mechanical contact - Option for reparability due to re-melting of the thermoplastic adhesive	- Suitable and low-cost bonders are not yet widely available - Reduced operating temperature depending on the selected adhesive
Ultrasonic welding [32,33]	- Fast production process	- Risk of some electrical components becoming damaged
Sewing [27]	- Process at room temperature - Commonly used in conventional textile production	- Contact requires additional encapsulation for fixation of yarns - Interposer with contacting pads is required
Snap fasteners [41]	- Process at room temperature - Contacts can also be external connectors	- Bulky, hard connectors reduce comfort - Parallel process with manual work steps - Interposer with pads for snap fasteners is required - Limited amount of interconnection contacts
Hook and loop strips [25,36]	- Process at room temperature - Commonly used system in textile production	- Conductive coating tends to peel off quickly - Low washing resistance - Tend to clog with textile fibres
3D printing with carbon filled filament [42]	- Fully customizable to contacted components	- High resistance of carbon-filled filaments - Low adhesion to textile substrates

**Table 3:** Parameters of the used hybrid conductive yarn.

Code	Composition	No. of Microwires	Microwire Diameter [µm]	Basic Yarn Count [Tex]	Yarn Diameter [µm]	Electrical Resistance [Ω/m]	Dry Tensile Strength [cN/Tex]	Dry Elongation [%]
I-COND art. 74 (Y08)	24% PES 76% Cu Ag	8	30	78	240	2.85	21.85	13.3

**Table 4:** Analysis of the maximum theoretical contact area consisting of hybrid conductive yarn microwires and the calculation of contact resistance.

Microwire Area [%]	Microwire Area [mm^2^]	Radius of the Appropriated Circular Area [µm]	Theoretical Contact Resistance R_c_ [mΩ]
12.30	0.095	174	0.189
14.54	0.113	189	0.174
19.12	0.148	217	0.152

**Table 5:** Parameters of the electrical contact created by the thermo-compression contacting technique.

R_c_ [mΩ]	Contact Area [µm^2^]	Radius of Circular Contact Area [µm]
4.5	166.4	7.27

## Data Availability

All data are in the paper.
